# A pipeline leakage detection method for boiler energy operation system using enhanced SVM-based acoustic emission technology

**DOI:** 10.1038/s41598-026-42769-5

**Published:** 2026-03-07

**Authors:** Tianlong Yuan, Xiaofei Zhang, Qian Zhang, Minghang Tan

**Affiliations:** 1 Liaoning Provincial Engineering Research Center for High-Value Utilization of Magnesite, Yingkou, 115014 China; 2 Liaoning Provincial Key Laboratory of Energy Storage and Utilization, Yingkou Institute of Technology, Yingkou, China; 3 State Grid Yingkou Electric Power Supply Company, Yingkou, China

**Keywords:** Pipeline, Leakage, Detection, Acoustic emission, Support vector machine, Energy science and technology, Engineering, Mathematics and computing

## Abstract

Pipeline leakage detection in boiler energy systems is essential for operational safety and efficiency, yet conventional techniques such as pressure-based and mass-balance methods often lack real-time performance and sensitivity to small leaks. Although acoustic emission (AE) technology offers dynamic, non-destructive monitoring, its practical application is hindered by noise interference and limited training samples under industrial conditions. This paper introduces an enhanced support vector machine (SVM) framework designed for robust AE-based leakage detection. The proposed approach integrates three key contributions: first, a multi-domain feature fusion strategy that combines time-domain and frequency-domain parameters for enhanced signal separability; second, a spectral sparsity-guided dynamic kernel selection mechanism that adaptively optimizes the model for varying signal characteristics; and third, a margin-based boundary sample weighting strategy that mitigates the influence of noise near the hyperplane. Experiments involving three leakage types—spot, fracture, and explosion tube—were conducted under both low-noise (40 dB) and high-noise (70 dB) conditions. The model achieved perfect classification (100% accuracy) under quiet settings, and maintained accuracies of 92.3%, 88.1%, and 85.4% for the respective leak types under noisy conditions, outperforming conventional SVM by 12–15%. These results demonstrate that the proposed framework significantly improves detection reliability in noisy, data-scarce environments, providing a practical tool for early leakage identification in industrial boiler systems. Future work will focus on adaptive noise modeling and online threshold learning to further enhance the framework’s robustness and adaptability in dynamic industrial settings.

**Supplementary Information:**

The online version contains supplementary material available at 10.1038/s41598-026-42769-5.

## Introduction

 The detection of pipeline leakages in boiler energy systems is critical for ensuring operational safety and efficiency. Existing leakage detection methods in industrial boilers can be broadly categorized into pressure-based, mass-balance, and acoustic techniques. Pressure-based approaches, such as the negative pressure wave (NPW) method, utilize transient pressure signals to locate leaks. Although NPW is cost-effective and accurate for abrupt leaks, its performance declines with gradual leaks and it often requires extensive sensor networks^1,2^. Mass-balance methods, which monitor discrepancies in fluid flow, typically lack real-time capability and sensitivity to minor leaks^3^. Acoustic emission (AE) technology has emerged as a dynamic, non-destructive alternative for leakage detection. In contrast to pressure-based methods, AE directly captures the elastic waves generated by material deformation or leakage events, enabling early detection without external excitation^4^. This technique offers high sensitivity, real-time monitoring, and notable adaptability to harsh environments, such as the high-temperature flue gas and ash erosion common in industrial boilers^5^. Consequently, AE has been widely adopted across various industries, including aerospace, petrochemical, and power generation.

Recent advances in machine learning (ML) have further strengthened AE-based leakage detection. Techniques such as convolutional neural networks (CNN), random forests, and twin support vector machine (TWSVM) have been applied to classify AE signals^6–8^. While deep learning models achieve high accuracy with large datasets, their performance often degrades with small samples-a common constraint in industrial settings. Although TWSVM improves multi-class classification, it is prone to overfitting. Similarly, wavelet-SVM combinations enhance feature extraction but at the cost of increased complexity. Despite the benefits of AE, its practical application for boiler pipeline leakage detection faces significant challenges. Industrial environments introduce substantial background noise, which can obscure leakage signals. Furthermore, traditional AE methods often contend with issues such as significant signal propagation attenuation, low sensitivity under noisy conditions, and limited signal coverage, making it difficult to reliably distinguish leakage signatures from interference. These challenges are exacerbated by the typical scarcity of labeled leakage data in industrial settings, which curtails the effectiveness of data-intensive deep learning models^9^. To address the limitations of standard SVM in multi-class problems and noisy environments, several advanced methods have been proposed. For instance, Sarkar et al. introduced a novel model combining twin random vector functional link networks with TWSVM, evaluating its efficiency and applicability^10^. Hazarika et al. proposed a fuzzy TWSVM method based on affinity; however, TWSVM often assigns equal classification weight to all samples, including those near the decision boundary, which can lead to classification errors or overfitting^11^. Gupta et al. developed an improved twin bounded SVM with Universum data for EEG classification^7^. Hazarika et al. also suggested a robust support vector quantile regression model using a truncated pinball loss function to reduce the impact of noise^12^. Other approaches, such as the adaptive neuro-fuzzy inference system (ANFIS) and least squares SVM (LSSVM), have shown excellent agreement with experimental measurements in certain applications^13^, and the Elman neural network has been used to accurately estimate biomass heating values^14^. More recently, hybrid deep learning models have demonstrated potential in handling complex temporal signals. For instance, Xue et al. proposed an LSTM-CNN-attention framework for evaporator tube leakage estimation, integrating temporal modeling with dual-path spatial feature extraction to achieve accurate leakage quantification under noisy conditions^15^. In a comprehensive review, Garcia et al. synthesized advancements in signal processing and hybrid physics-data models for fault diagnosis, highlighting their promise alongside persistent challenges such as data scarcity and limited explainability^16^. Despite their power, the data-hungry nature and computational complexity of these deep learning methods can be prohibitive for real-time applications with limited samples. This gap motivates the present work, which aims to develop a robust, sample-efficient framework that leverages the strengths of SVMs while enhancing their adaptability and noise resilience through intelligent feature engineering and model adjustment.

This paper addresses these needs by proposing an enhanced SVM framework optimized for AE signal classification under limited samples and noisy conditions. While kernel-adaptive and weighted SVMs exist in the literature, our framework introduces a synergistic combination of three novel components, specifically tailored for AE-based leak detection:① Multi-domain feature fusion: We systematically integrate both time-domain (e.g., Range, Duration, RMS) and frequency-domain (e.g.,Energy) parameters into an 8-dimensional vector to provide a more comprehensive representation of leak signals, addressing a limitation of traditional single-domain approaches.② Spectral sparsity-guided dynamic kernel selection: Unlike pre-defined or manually selected kernels, our method automatically chooses between linear and nonlinear kernels based on the Spectral Sparsity Index (SSI) of the signal, ensuring the model’s complexity aligns with the signal’s inherent separability.③ Margin-based boundary sample weighting: We introduce a sample-specific weighting scheme that prioritizes samples near the decision boundary, which are most vulnerable to noise corruption. This strategy, distinct from generic weighting methods, directly tackles a core challenge in noisy industrial environments. The novelty of this work lies not only in these individual components but also in their integration into a unified, robust, and adaptive framework that effectively addresses the specific limitations of AE signal classification in practical boiler systems. A comparison of different machine learning approaches for AE-based leakage detection is summarized in Table 1.

**Table 1:** Comparison of different machine learning approaches for AE-based leakage detection.

Method	Typical data requirements	Pros	Cons	Typical accuracy (in noisy, small-sample scenarios)
Traditional ML (e.g., SVM)	Small to moderate	Good performance with small samples; Computationally efficient	Sensitive to noise and feature engineering; Limited adaptability to complex patterns	Moderate (e.g., ཞ70–75% as baseline in this study)
Deep learning (e.g., CNN)	Large	High accuracy with sufficient data; Automatic feature extraction	Prone to overfitting with small samples; Computationally intensive	Low to moderate (with limited data)
Proposed enhanced SVM	Small	Robust to noise; Handles small samples effectively; Adaptive kernel selection	Computational overhead for kernel selection	High (85%−92%, as demonstrated in this study)

## Basic principle of detecting pipeline leakages

### Principle of SVM detection for pipeline leakages

**Fig. 1:**
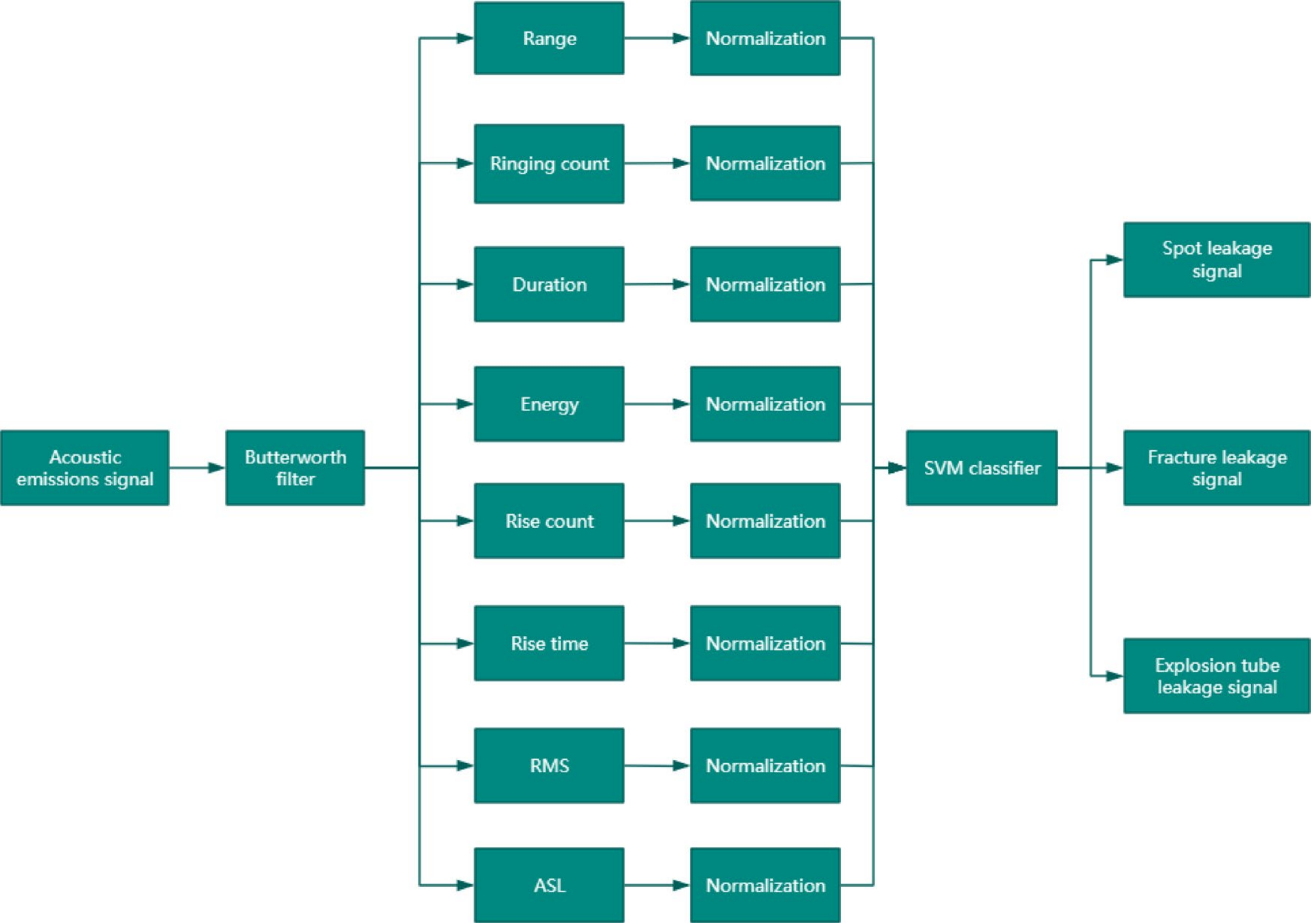
Flowchart of AE testing for pipeline leakages.

The application of Support Vector Machine (SVM) for boiler pipeline leakage detection follows a systematic workflow, as illustrated in Fig. 1. The process consists of the following key steps: (1) AE signal preprocessing: The acquired AE signals, which are often contaminated by strong background noise, are first denoised using a Butterworth filter. This step aims to restore and enhance the useful signal components. (2) Extraction of AE characteristic parameters: Based on the standard definitions of AE signal parameters, a set of discriminative features is extracted from the pre-processed signals. (3) Pattern Recognition for Early Leakage Signals: The extracted feature parameters are combined to form an eight-dimensional feature vector, integrating both linear and non-linear characteristics. Each dimension of the feature vector is normalized and then fed into an SVM classifier to enable the automatic identification and classification of leakage-related AE signals. This methodology leverages the robust pattern recognition capabilities of SVM to distinguish between different types of leakage signals based on their fused acoustic emission features.

### Principle of AE detection

AE testing is a dynamic non-destructive testing method based on the detection of transient elastic waves generated by the rapid release of energy within a material. When pipeline leakage occurs, the escaping medium produces stress waves that propagate along the pipe wall. The fundamental principle of AE detection involves using highly sensitive piezoelectric sensors to capture these waves, followed by advanced signal processing to extract characteristic parameters that reveal the nature and location of the leakage source^17^. The most distinctive feature of AE technology, compared to other non-destructive testing methods, is its passive nature. Unlike active techniques that require external excitation, AE signals originate directly from the defect itself, making this method particularly sensitive to dynamic changes in material structure. This inherent sensitivity, combined with the universal acoustic emission characteristics of most engineering materials, allows AE monitoring to transcend material limitations and provide continuous, real-time assessment of structural integrity. In this study, we employ waveform parameter analysis to process and characterize AE signals from boiler pipeline leaks. This approach involves preliminary processing and systematic organization of measured AE signals through a set of simplified waveform parameters. Common AE parameters include ringing count, duration, and threshold voltage. Building on this foundation, we select eight key parameters to form a comprehensive feature vector for subsequent analysis and pattern recognition using SVM, enabling effective discrimination between different leakage types.

### SVM

#### Principle of SVM

SVM is a general-purpose learning model developed based on statistical learning theory. By minimizing structural risk, it effectively estimates the actual risk, thereby enhancing the algorithm’s generalization capability and serving as an effective tool for limited-sample learning. With technological advances, more advanced or variant models have been derived from SVM, including TWSVM and nonparallel hyperplane SVM (NHSVM). When classifying an unknown sample, the TWSVM classifier computes the distances from the sample point to two nonparallel hyperplanes and assigns it to the class corresponding to the closer hyperplane. However, TWSVM has a notable limitation: inconsistency between the training and prediction phases. During prediction, the classifier compares the distances to both hyperplanes, assigning the sample to the class with the nearer hyperplane. Yet, during training, such a comparative mechanism is absent—only the distance to the opposite-class hyperplane is considered^18^. In contrast, NHSVM constructs two nonparallel hyperplanes by solving a quadratic programming (QP) problem, under the principle that each hyperplane should be close to samples of its own class and appropriately distant from the other class. However, a discrepancy exists between the distance metric used in this partitioning rule and the one formulated in the optimization problem. Practical applications have revealed that such a partitioning strategy tends to be inefficient. The classical SVM approach obtains a pair of parallel hyperplanes by maximizing the margin and selects the median hyperplane for decision-making. The core idea is to identify the optimal separating hyperplane—either in the original input space or in a projected high-dimensional space^10^, correctly separates the two classes while maximizing the classification margin between them^19^.

For a linearly separable sample set$({x_i},{y_j})$, $i=1,2, \cdots n$, ${\mathbf{x}} \in {{\mathbf{R}}^d}$, and ${y_j} \in \left\{ { - 1,+1} \right\}$. The classification line equation can be expressed as: ${{\mathbf{w}}^T}{\mathbf{x}}+b=0$. The equation is normalized so that the sample set meets the constraint conditions, as given by Eq. (1).

1 $${y_i}({{\mathbf{w}}^T}{x_i}+b) - 1 \geqslant 0,\: i=1,2, \cdots n$$

where the sample that generates the equality is called the support vector. The classification gap of the two types of samples is given by: ${2 \mathord{\left/ {\vphantom {2 {\left\| {\mathbf{w}} \right\|}}} \right. \kern-0pt} {\left\| {\mathbf{w}} \right\|}}$. Therefore, under the above constraints, the optimal classification surface problem can be expressed as a constrained optimization problem by finding the minimum value of the function, as given by Eq. (2).

2 $$\varphi ({\mathbf{w}})=\frac{1}{2}{\left\| {\mathbf{w}} \right\|^2}=\frac{1}{2}({{\mathbf{w}}^T}{\mathbf{w}})$$

For this purpose, the Lagrange function can be defined as Eq. (3).

3 $$L({\mathbf{w}},b,\alpha )=\frac{1}{2}{{\mathbf{w}}^T}{\mathbf{w}} - \sum\limits_{{i=1}}^{n} {{\alpha _i}\left[ {{y_i}({{\mathbf{w}}^T}{{\mathbf{w}}_i}+b) - 1} \right]}$$

where ${\alpha _i} \geqslant 0$is the Lagrange coefficient. The original problem is transformed into the dual problem of convex quadratic programming, as given by Eq. (4).

4 $$\left\{ \begin{gathered} \max \left[ {\sum\limits_{{i = 1}}^{n} {\alpha _{i} - \frac{1}{2}\sum\limits_{{i = 1}}^{n} {\sum\limits_{{j = 1}}^{n} {\alpha _{i} \alpha _{j} y_{i} y_{j} (x_{i} ^{T} x_{j} )} } } } \right] \hfill \\ s.t.\,\,\,\,\,\,\,\,\alpha _{i} \ge 0,i = 1,2, \cdots ,n \hfill \\ \sum\limits_{{i = 1}}^{n} {\alpha _{i} y_{i} = 0} \hfill \\ \end{gathered} \right.$$

This is a quadratic function mechanism problem under inequality constraints, and there is a unique optimal solution. If ${\alpha _i}^{*}$ is the optimal solution, then: ${{\mathbf{w}}^*}=\sum\limits_{{i=1}}^{n} {{\alpha _i}^{*}{y_i}} {{\mathbf{x}}_i}$. Moreover, ${\alpha _i}^{*}$is a support vector, and the weight coefficient vector of the optimal classification surface is a linear combination of support vectors. Furthermore, *b* can be solved by the constraint: ${y_i}({{\mathbf{w}}^T}{x_i}+b) - 1=0$. The optimal classification function obtained is given by Eq. (5).

5 $$f({\mathbf{x}})=sgn[{({{\mathbf{w}}^*})^T}{\mathbf{x}}+{b^*}]=sgn(\sum\limits_{{i=1}}^{n} {{\alpha _i}^{*}{y_i}{{\mathbf{x}}_i}^{*}{\mathbf{x}}+{b^*}} )$$

where $\mathrm{sgn} (\,)$ is a symbolic function.

For linearly inseparable samples, the input vectors can be mapped into a high-dimensional feature space, where an optimal hyperplane is determined to partition the feature space into two regions. By introducing a kernel function, the inner product operation in the high-dimensional feature space can be replaced by the kernel function computed in the original input space, which means: $K(x_{i} ,x_{j} ) = \varphi (x_{i} ) \cdot \varphi (x_{j} )$. This helps avoid the dimensional disaster. When two types of points cannot be completely separated by a hyperplane (only a few points are mis-divided), the relaxation variable ${\xi _i}$ and penalty parameter *C* can be introduced to transform the problem of finding the optimal classification surface into^20^ Eq. (6).

6 $$\hbox{min} \psi (w,\xi )=\frac{1}{2}{{\mathbf{w}}^T}{\mathbf{w}}+C\sum\limits_{{i=1}}^{n} {{\xi _i}}$$

The optimal classification function can be expressed as Eq. (7).

7 $$f({\mathbf{x}})=sgn[{({{\mathbf{w}}^*})^T}{\mathbf{x}}+{b^*}]=sgn(\sum\limits_{{i=1}}^{n} {{\alpha _i}^{*}{y_i}{{\mathbf{x}}_i}^{*}{\mathbf{x}}+{b^*}} )$$

The SVM algorithm was originally developed for binary classification tasks. To address multi-class problems, it is necessary to construct a suitable multi-class classifier. Currently, there are two primary approaches for building multi-class SVMs. The first is the direct method, which modifies the objective function directly by integrating the parameters of multiple classification hyperplanes into a single optimization problem. This approach solves the multi-class classification in one step^21^. Although conceptually straightforward, this method suffers from high computational complexity, is difficult to implement, and is generally only suitable for small-scale problems. The second approach is the indirect method, which combines multiple binary classifiers to form a multi-class classifier. Common strategies include the “one-versus-rest” and “one-versus-one” methods^22^.

In the one-versus-rest approach, samples from one class are treated as the positive class, and all other samples are grouped as the negative class. For an *n*-class problem, *n* SVM classifiers are constructed. During testing, the class label of a test sample is assigned to the class whose classifier yields the highest output value. A drawback of this method is the large training set size for each binary classifier, which complicates training and may lead to unbounded generalization error.

In the one-versus-one method, a separate SVM classifier is trained for every pair of classes, requiring *n(n-1)/2* classifiers in total for *n* classes. During prediction, each binary classifier votes on the test sample, and the class with the most votes is selected as the final prediction. The LIBSVM toolbox used in this study implements multi-class classification based on this one-versus-one strategy^23^.

While the traditional SVM formulation, as described in Eq. (1) to (7), provides a robust theoretical foundation for binary classification, its performance in practical industrial applications—such as boiler pipeline leak detection—is often limited by two major challenges: (1) strong background noise that corrupts acoustic emission features and introduces ambiguous samples near the decision boundary, and (2) the difficulty of pre-selecting a single kernel function suitable for all types of leak signals, which often exhibit diverse spectral characteristics. To address these limitations, we propose an enhanced SVM framework incorporating three key modifications: multi-domain feature fusion (Sect. 2.3.3.1), dynamic kernel selection (Sect. 2.3.3.2), and a boundary sample weighting strategy (Sect. 2.3.3.3). The latter two enhancements are implemented through direct modifications to the standard SVM optimization objective.

#### Least squares support vector machine

A mathematically grounded variant of the SVM approach is the least squares support vector machine (LSSVM). Compared with artificial neural network (ANN) algorithms, LSSVM offers advantages such as faster convergence, fewer parameters to adjust, and a reduced tendency to overfit. By replacing the quadratic programming problem in standard SVM with a system of linear equations, LSSVM simplifies the optimization process and enhances computational efficiency. The model is trained using $\left\{ {\left( {{x_1},{y_1}} \right),\left( {{x_2},{y_2}} \right),...\left( {{x_N},{y_N}} \right)} \right\}$ given dataset and a nonlinear kernel function, as formulated in Eq. (8)^24^.

8 $$g\left( x \right)=b+\phi \left( x \right),\omega$$

where g(x), $\phi$, and *x* are the output, nonlinear approximation function, and input variable, respectively, and *b* and $\omega$ are the bias and weight terms, respectively.

The real-world performance of the proposed SVM-based pipeline leak detection model may be influenced by high levels of background noise and variations in signal characteristics, particularly in scenarios involving limited sample data or constrained computational resources. In contrast, multi-parameter mathematical models account for multiple interacting factors, which can potentially enhance prediction accuracy and robustness. Such models are especially advantageous when sufficient data and computational power are available, enabling more detailed and potentially more reliable predictions.

#### Enhanced SVM framework and dynamic kernel selection and boundary sample weighting algorithm for leak detection

**Fig. 2:**
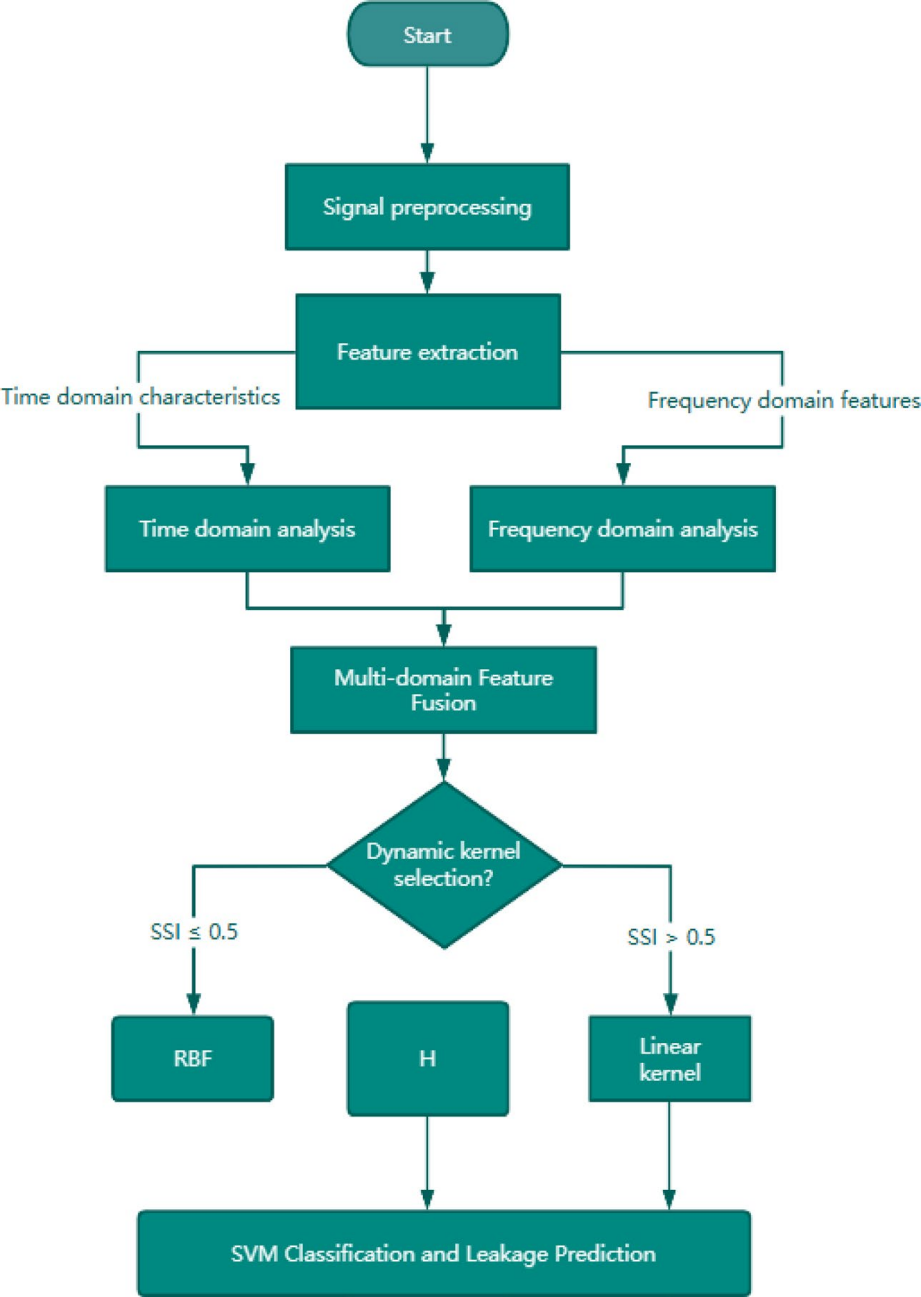
Schematic of the enhanced SVM framework for pipeline leakage detection.

To address the limitations of traditional SVM in classifying noisy and non-stationary leak signals, this study introduces an enhanced SVM framework that integrates multi-domain feature fusion, adaptive kernel selection, and boundary sample weighting. Figure 2 schematically illustrates the proposed classification framework, demonstrated through three representative classification scenarios. The workflow combines multi-domain feature extraction, spectral sparsity-guided dynamic kernel selection, and margin-based sample weighting to achieve robust classification under noisy conditions.

**Fig. 3:**
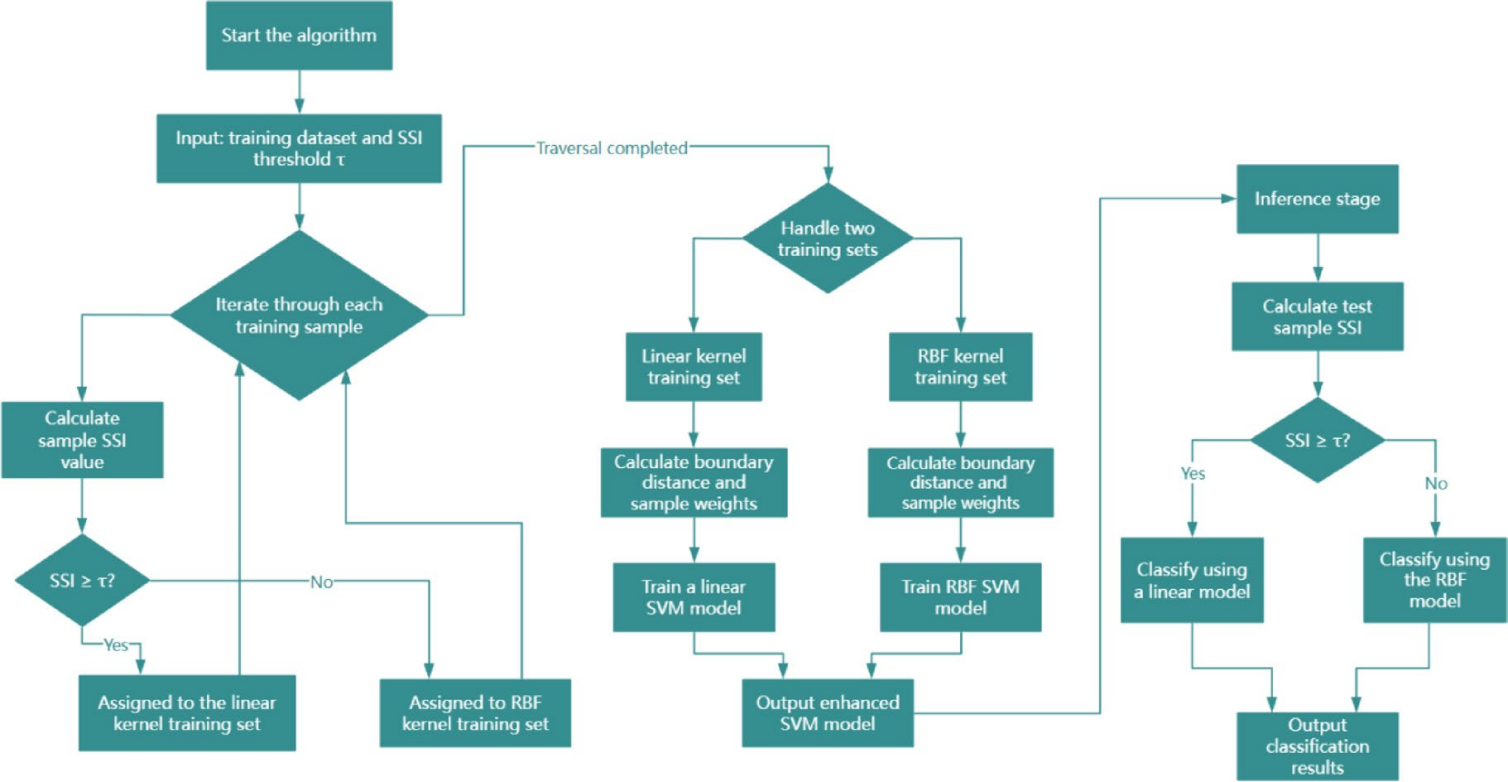
Flowchart of the dynamic kernel selection and boundary sample weighting algorithm in the enhanced SVM framework.

The overall workflow of the proposed enhanced SVM framework particularly the dynamic kernel selection and boundary sample weighting algorithms is illustrated in the flowchart in Fig. 3. The process begins with inputting training samples, followed by multi-domain feature extraction to form a comprehensive feature vector. The spectral sparsity index is then computed for each sample. Based on a precomputed threshold of this index, a dynamic decision mechanism selects the appropriate kernel model (either linear or RBF) for the subsequent training phase. During training, a boundary sample weighting strategy is applied, which calculates the margin distance of each sample and assigns corresponding weights. This approach enhances the influence of samples near the decision boundary while mitigating the impact of noise. Finally, the trained enhanced SVM model is deployed for leak-type classification. As shown in Fig. 3, this structured methodology ensures adaptive model selection and improves the robustness of the final classification under noisy conditions.

##### Multi-domain feature fusion

Leak-induced acoustic signals exhibit transient and nonlinear characteristics, necessitating comprehensive feature characterization. To this end, we construct an 8-dimensional feature vector by integrating parameters from both the time and frequency domains. The time-domain features comprise: range, ringing count, duration, rise count, root mean square (RMS), and average signal level (ASL). The frequency-domain is represented by energy. The names and detailed interpretations of these characteristic parameters are summarized in Table 2.

**Table 2:** Parameters of AE signals.

Parameter name	Implication
Time of arrival	The time an acoustic emission wave reaches the sensor
Channel number	Detect the channel number corresponding to the acoustic emission wave
Range	The maximum amplitude value of the signal waveform
Ringing count	The number of oscillations over the threshold signal can be divided into total number and count rate
Duration	The interval between when the signal first crosses the threshold and when it finally reaches it
Energy	The area below the envelope of signal detection can be divided into total number and count rate
Rise count	The number of oscillations the signal first crosses the threshold to its maximum amplitude
Rise time RMS ASL	The interval between the first time the signal crosses the threshold and the maximum amplitude The root-mean-square value of the signal during the sampling time The mean of the signal level during the sampling time

The Range, defined as the difference between the maximum and minimum amplitudes within a time window, reflects the signal’s dynamic range and is calculated using Eq. (9).

9 $$R=\hbox{max} \left( {xi} \right) - \hbox{min} \left( {xi} \right),i=1,2,...,N.$$

The Energy-based feature is calculated by Eq. (10).

10 $$RMS=\sqrt {\frac{1}{N}\sum\limits_{{i=1}}^{N} {{x_i}^{2}} }$$

The Average Signal Level (ASL), representing the mean amplitude over a time window, is given by Eq. (11).

11 $$ASL=\frac{1}{N}\sum\limits_{{i=1}}^{N} {\left| {{x_i}} \right|}$$

Total spectral energy within the leak-sensitive band is computed on via discrete Fourier transform (DFT) as Eq. (12).

12 $$E=\sum\limits_{{k={k_1}}}^{{{k_2}}} {{{\left| {X({f_k})} \right|}^2}}$$

where $X({f_k})$ is the DFT coefficient at frequency${f_k}$.

Range and Ringing Count characterize transient leak-induced pulses. Rise Time and Rise Count help distinguish abrupt leak onsets from gradual mechanical vibrations. Energy and RMS collectively quantify the intensity of leakage signals, whereas Duration aids in filtering out short-duration noise spikes. The fusion of these features enhances the separability between leak signals and interference by leveraging their complementary information.

The selection and fusion of these eight parameters are grounded in the distinct physical processes underlying each leakage type, enabling the feature vector to capture complementary aspects of the acoustic emission signals.

Spot leakage typically results from a sudden, localized breach (e.g., a pinhole), causing a short-duration, high-amplitude pressure release. This transient burst is characterized by a high Range (significant amplitude swing), a moderate Ringing Count (limited oscillations), and a relatively shorter Duration. Its broadband frequency spectrum, as observed in Fig. 6, leads to a widely distributed Energy profile, while the RMS and ASL values are moderate, reflecting the signal’s intensity and average level during the brief event.

Fracture leakage involves sustained fluid escape through a propagating crack, generating a continuous, oscillatory signal. This process manifests in distinctly high values for Duration and Ringing Count, indicating prolonged activity and numerous oscillations. The narrowband, high-frequency concentration of energy (Fig. 7) results in a high but focused Energy value. The RMS and ASL are typically lower than in explosion leaks but may show less fluctuation due to the signal’s more stable nature.

Explosion-induced leakage is a consequence of a violent, catastrophic rupture, releasing intense acoustic energy instantaneously. This physics leads to the highest recorded values for Range, RMS, and total Energy among the three types, directly quantifying the massive amplitude and power of the event. The low-frequency-dominated broadband spectrum (Fig. 8) further shapes its unique spectral signature. While the Duration might be short like a spot leak, the extreme amplitudes differentiate it.

Parameters like Rise Time and Rise Count aid in distinguishing the signal onset sharpness, potentially different between an abrupt rupture (fast rise) and a crack’s initial phase. By fusing these time-domain and frequency-domain features, the model leverages a comprehensive signature that encodes the leakage physics, thereby enhancing separability, especially under noise where single-domain representations may become ambiguous.

##### Dynamic kernel selection mechanism

To adaptively balance model complexity and generalization capability, we introduce a spectral-guided kernel selection strategy.

The spectral sparsity index ($SSI$), defined by Eq. (13), is introduced to quantify the normalized sparsity of the signal spectrum as a measure of separability.

13 $$SSI=\frac{{{{\left\| {X\left( {f\left( k \right)} \right)} \right\|}_\infty }}}{{{{\left\| {X\left( {f\left( k \right)} \right)} \right\|}_2}}}$$

Where${\left\| {X\left( {f\left( k \right)} \right)} \right\|_\infty }$is the maximum spectral amplitude of the signal, and${\left\| {X\left( {f\left( k \right)} \right)} \right\|_2}$is the L2-norm of the spectral amplitude vector, $SSI$is the total number of frequency components in the spectrum.

When $SSI \geqslant 0.65$, it indicates sparse spectra with dominant frequency components, suggesting the signals are more likely to be linearly separable. When $SSI \leqslant 0.65$, it indicates broadband or multi-component spectra, requiring nonlinear separation. Precompute $SSI$ during feature extraction and train two SVM models offline using identical training data.

The threshold θ for spectral sparsity index (SSI) is determined through a data-driven optimization procedure. Specifically, we perform a grid search over a candidate range θ∈[0.1, 0.9] with a step size of 0.05 on the training set. For each candidate θ, the training samples are split into two subsets according to SSI ≥ θ (assigned to linear kernel) and SSI < θ (assigned to RBF kernel). A5-fold cross-validation accuracy is computed for the combined kernel strategy. The θ value that yields the highest average validation accuracy is selected. In this study, the optimal θ was found to be 0.65, which effectively separates signals with dominant spectral components (linear-kernel suitable) from those with broad or multi-component spectra (nonlinear-kernel needed). This systematic selection ensures that the kernel assignment adapts to the inherent separability of the acoustic emission signals rather than relying on empirical heuristics.

##### Boundary sample weighting strategy

The performance of SVM can be degraded by noise-corrupted samples located near the decision hyperplane. This issue can be mitigated through a margin-based sample weighting strategy.

For each training sample ${x_i}$, its distance to the decision hyperplane is calculated using Eq. (14).

14 $${d_i}=\frac{{\left| {{w^T}\phi \left( {{x_i}} \right)+b} \right|}}{{\left\| w \right\|}}$$

where *w* and *b* are the weight vector and bias term of the trained SVM hyperplane, respectively. The distance${d_i}$quantifies the confidence of the classification for sample${x_i}$;a smaller$\left| {{d_i}} \right|$indicates the sample is closer to the decision boundary and thus more vulnerable to being misclassified under noise.

Samples with smaller margin distances (${d_i}<\delta ,\delta =0.15$) are assigned higher weights, as defined in Eq. (15).

15 $${\alpha _i}=\frac{1}{{1+\exp \left( { - \gamma \left( {{d_i} - \delta } \right)} \right)}}$$

where $\gamma$ is a scaling parameter that controls the steepness of the weighting function. This formulation assigns a weight ${\omega _i}$ close to 1 (standard penalty) to high-confidence samples far from the boundary, and a weight significantly greater than 1 to critical, low-confidence samples near the boundary.

The SVM objective function is reformulated as shown in Eq. (16).

16 $$\mathop {\hbox{min} }\limits_{{w,b}} \frac{1}{2}{\left\| w \right\|^2}+C\sum\limits_{{i=1}}^{n} {{\alpha _i}{\xi _i}}$$

This objective function modifies the standard soft-margin SVM formulation (Eq. (6)) by incorporating the sample-specific weight${\omega _i}$. This means misclassifying a high-weight boundary sample incurs a greater penalty$\left( {{\omega _i}C} \right)$, effectively forcing the hyperplane to be positioned more robustly against such ambiguous points.

## Experiments

Three types of simulated leakage AE signals‒spot leakage, fracture leakage, and explosion-induced leakage were acquired from the pipeline of a laboratory-scale waste heat boiler. Based on analyses of the time-domain waveforms and frequency spectra of these AE signals, an SVM model was applied to characterize the leakage signatures. Eight discriminative indices amplitude, ringing count, duration, energy, rise count, rise time, RMS, and average ASL were selected to form a feature vector as input to the SVM. This approach was employed to diagnose pipeline leakage and validate the feasibility of using SVM for AE signal classification.

### Constitution of experimental environment and system

**Fig. 4:**
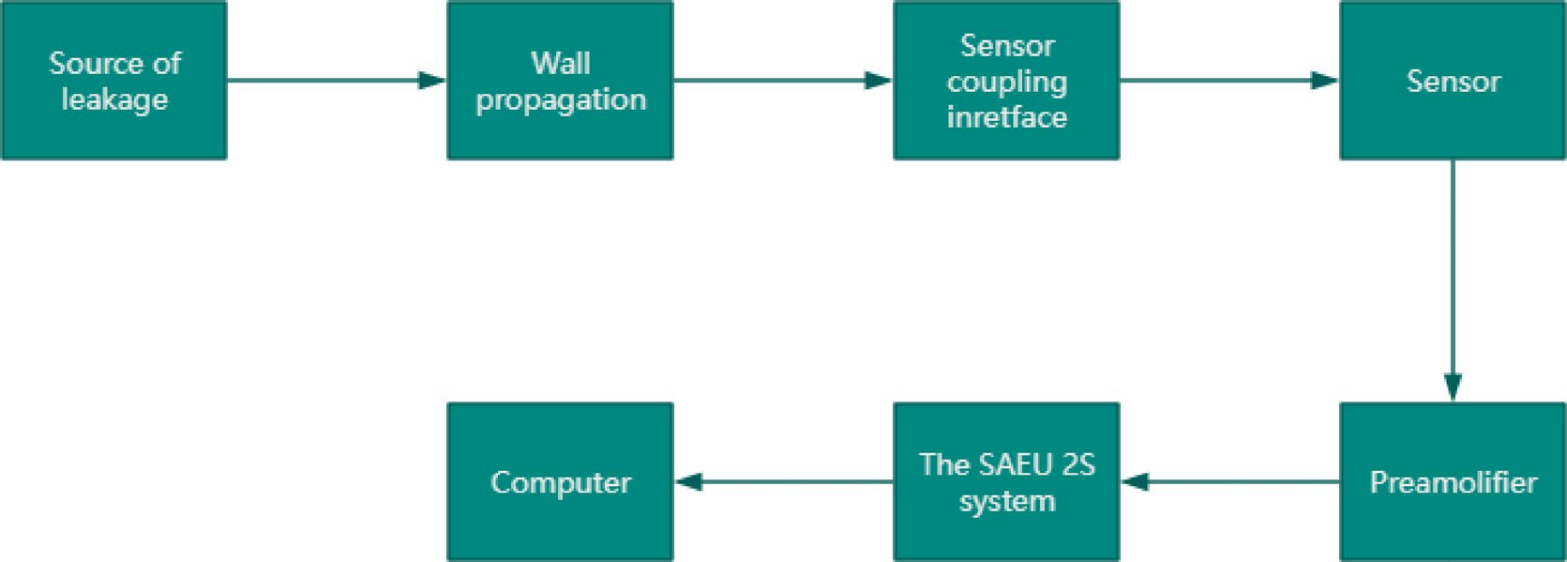
Flow chart of AE monitoring apparatus and ancillary measurement devices.

The experimental investigation was conducted under controlled laboratory conditions with significantly attenuated ambient noise levels (approximately 40 dB), representing a 30 dB reduction compared to typical industrial environments where noise levels generally exceed 70 dB. To enhance signal fidelity, a preloaded coupling mechanism was employed between the piezoelectric sensor and the pipeline surface, minimizing signal attenuation due to impedance mismatch. The AE acquisition system comprising piezoelectric transducers (Model XYZ, frequency response: 20­400 kHz) and a 16-bit data acquisition module (sampling rate: 1 MHz)‒was specifically configured for leakage detection in pressurized pipeline systems. The complete experimental setup, including both the AE monitoring apparatus and ancillary measurement instruments, is schematically illustrated in Fig. 4.

#### AE instrument

The AE measurements were performed using an SAEU2S system (Beijing Sonhua Technology Co., Ltd., China).This instrument provides 40 AE channels, meeting all experimental requirements for this study. The AE signal processing chain consists of the following stages: signals are first amplified by a pre-amplifier with specified filter frequencies, then digitized by a high-speed analog-to-digital (A/D) converter. The digitized signals are processed by the main processor, which extracts, analyzes, and computes conventional characteristic parameters. Finally, the processed data are recorded and displayed^25,26^.

#### Sensor

The SR150M sensor (Physical Acoustics Corp., USA) operates within a frequency range of 20–400 kHz, making it suitable for detecting acoustic emission signals from various metallic materials.

#### Experimental pipeline

The experimental setup consisted of six parallel pipeline rows with an inter-pipeline spacing of 45 cm. The pipelines had a total length of 70 m and a diameter of 9.58 cm.

### Selection of the parameters for the experimental equipment

The equipment parameters were determined through iterative calibration and are summarized in Table 3.

**Table 3:** The main parameters of AE device.

Device parameter name	Parameter value	Device parameter name	Parameter value
Sampling length	20,000 µs	Parameter threshold	40 db
Sampling frequency	2000 kHz	Advance gain	40 db
Parameter interval	2000 µs	Filter	20**–**400 k
Lockout time	2000 µs	Soft threshold	70 db
Software lockout time	1000 µs	Velocity of sound	4.7 km/s
Waveform threshold	40 db	Locate the radius of the concentration area	15 mm

### Experimental scheme

**Fig. 5:**
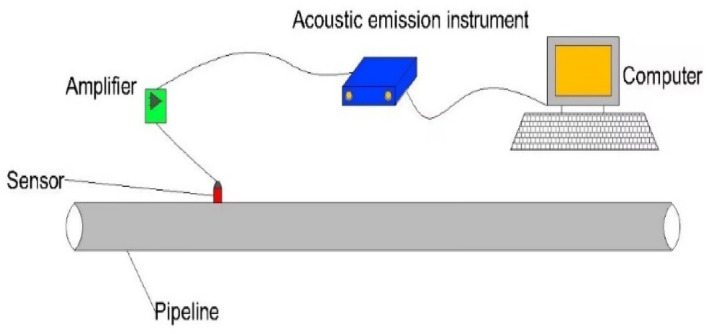
Schematic of pipeline leak detection using AE method.

Experiments were conducted under both ideal laboratory conditions and simulated industrial noise environments. Under controlled laboratory settings, background noise was maintained below 40 dB, while Gaussian noise (20­100 kHz) was introduced to simulate on-site industrial conditions and evaluate system robustness. Three types of leakage acoustic AE signals‒spot leakage, fracture leakage, and explosion-induced leakage were generated from the pipeline of a laboratory-scale waste heat boiler. Time-domain waveforms and frequency spectra of these AE signals were analyzed, and SVM was employed to process the characteristic parameters of leakage signals. Eight discriminative indices amplitude, ringing count, duration, energy, rise count, rise time, RMS, and ASL were selected to form a feature vector as input to the SVM model, which served as the diagnostic tool for pipeline leakage classification. The results confirm the applicability of SVM for AE signal classification. A schematic diagram of the AE-based pipeline leak detection methodology is presented in Fig. 5.

#### Time-frequency comparative analysis of AE signals

**Fig. 6:**
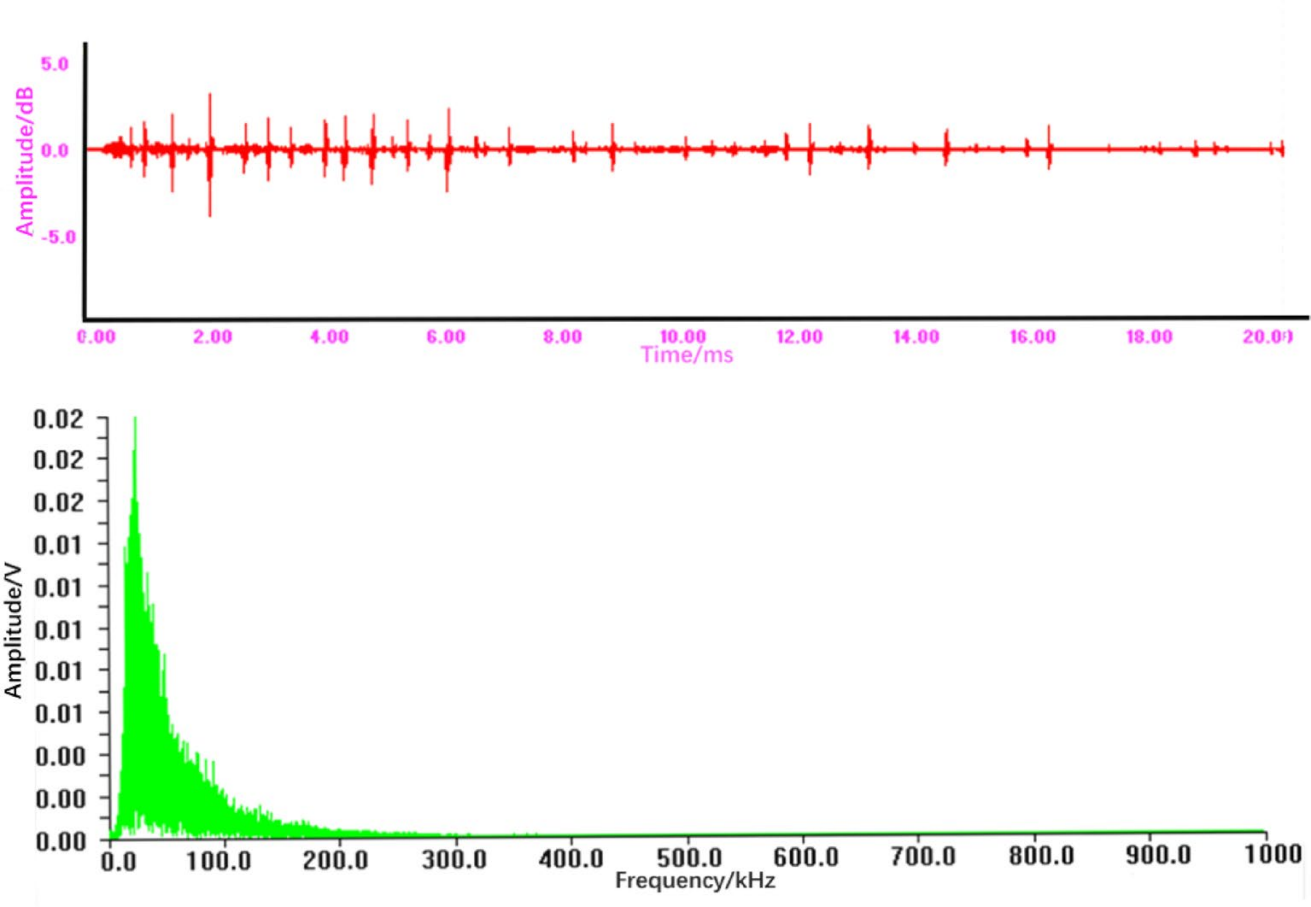
Time-domain waveform and frequency spectrum of the acoustic emission signal from a spot leakage.

**Fig. 7:**
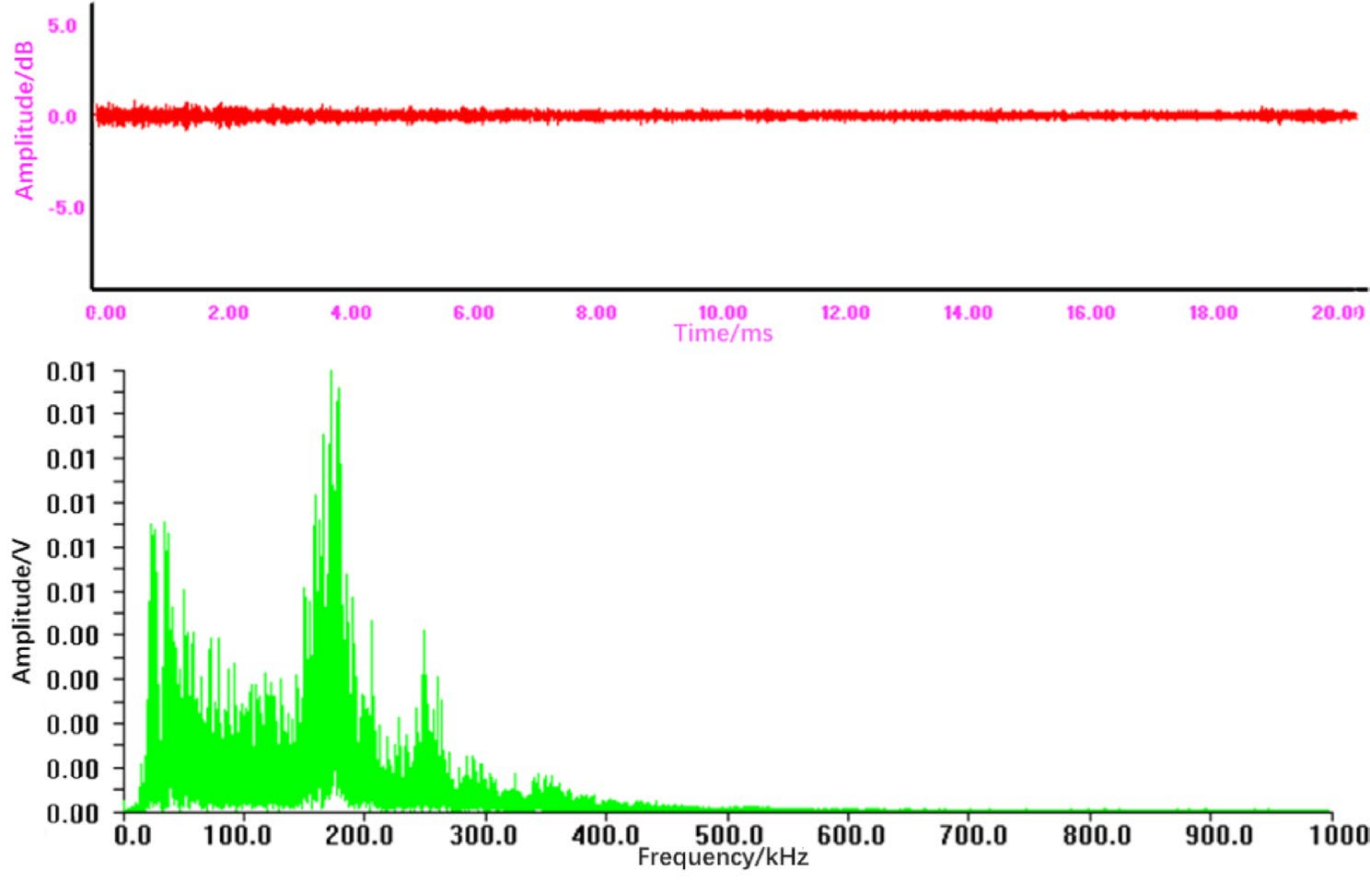
Time-domain waveform and frequency spectrum of the acoustic emission signal from a fracture leakage.

**Fig. 8:**
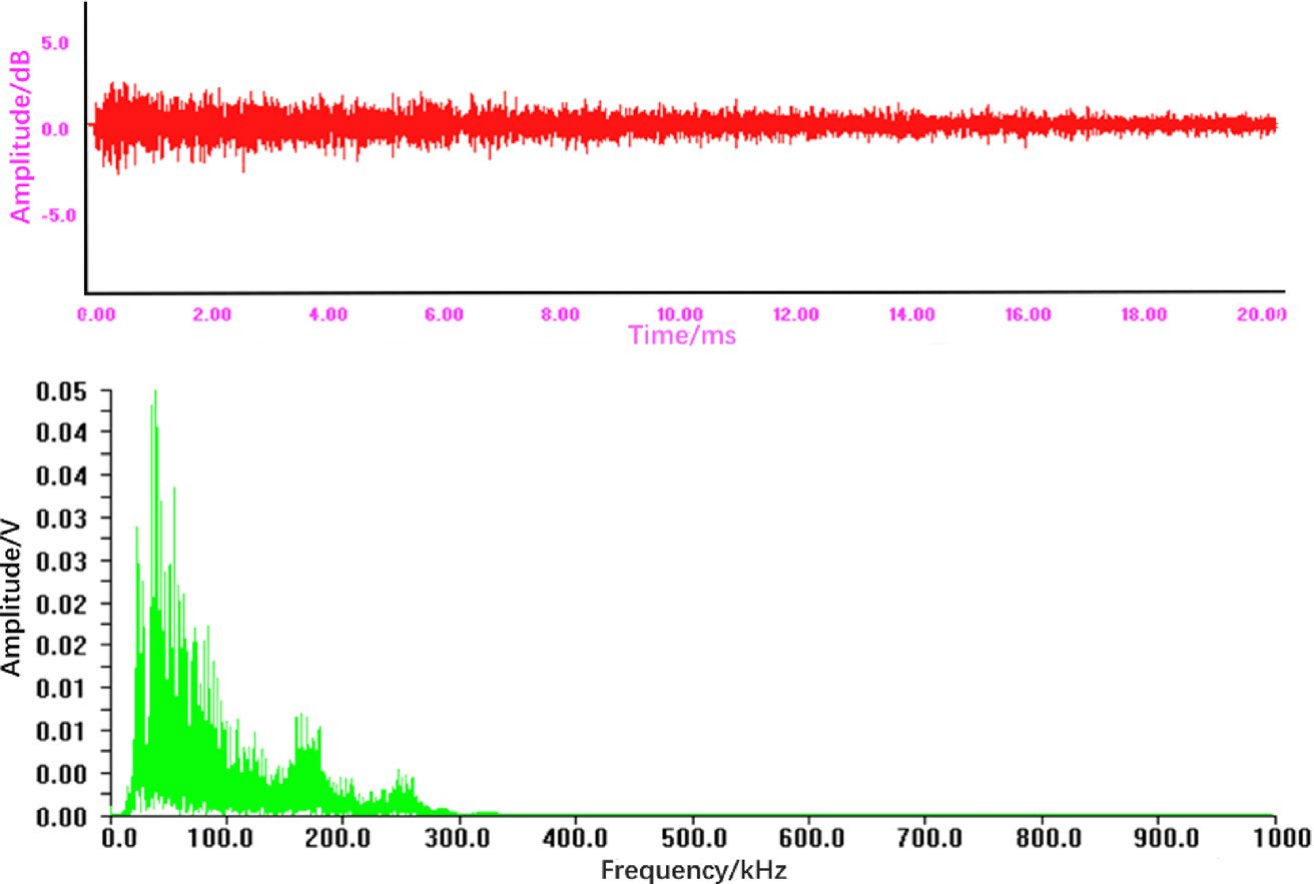
Time-domain waveform and frequency spectrum of the acoustic emission signal from an explosion tube leakage.

Figure 6 shows the time-domain waveform and frequency spectrum of the AE signal from a spot leakage. Figure 7 shows the time-domain waveform and frequency spectrum of the AE signal from a fracture leakage, whereas Fig. 8 shows the time-domain waveform and frequency spectrum of the AE signal from an explosion tube leakage.

From Fig. 6, it can be seen that:

1. The time-domain signal exhibits a transient, burst-like pattern characterized by rapid onset, high amplitude, and significant fluctuations with no discernible periodicity, which is typical for a sudden pressure release.
2. The corresponding frequency spectrum demonstrates a broadband characteristic, with signal energy distributed across a wide frequency range of 10­140 kHz. The maximum amplitude observed is approximately 0.02 V. This broadband nature aids in distinguishing spot leaks from mechanical noise, which often occupies narrower frequency bands.

These observed characteristics—transient burst in time domain and broadband spectrum—are quantitatively captured by features such as high Range, moderate Ringing Count, and a broadly distributed Energy profile.

From Fig. 7, it can be seen that:

1. In contrast to the spot leakage, the time-domain waveform presents a continuous, oscillatory pattern with relatively stable amplitude and periodic fluctuations before and after the main event, indicative of a sustained leak through a crack.
2. The frequency spectrum is notably narrowband, with dominant frequency components tightly concentrated within the high-frequency range of 150­190 kHz. The maximum amplitude here is lower, at approximately 0.01 V. This concentrated, high-frequency spectral signature is a key identifier for fracture-type leakage.

The sustained, oscillatory nature of the leak is directly reflected in significantly higher values of Duration and Ringing Count, while its narrowband concentration results in high Energy within a specific frequency band.

From Fig. 8, it can be seen that:

1. The time-domain signal shows an intense, high-energy burstwith the largest amplitude among the three leakage types and substantial fluctuations, reflecting the violent nature of a tube explosion.
2. The frequency spectrum is broadband but predominantly concentrated in the low-frequency region of 10­100 kHz. A dominant peak is observed with a maximum amplitude of approximately 0.05 V, which is significantly higher than that of the other leak types. The high energy and distinct spectral profile make explosion signals the most salient among the classes.

The violent release of energy manifests as the highest recorded values for Range, RMS, and total Energy, with the low-frequency-dominant broadband spectrum further distinguishing its feature signature.

#### Sample data classification processing

Eight indexes including amplitude, ringing count, duration, energy, rise count, rise time, RMS, and ASL were selected and input to SVM for training. The three leakage types are labeled as follows: 1 for spot leakage, 2 for fracture leakage, and 3 for explosion-induced leakage. A total of 90 experimental samples were collected, with 30 samples per leakage type. To ensure statistical significance and robustness in classification, the dataset was randomly divided into a training set and a testing set with a ratio of 2:1 (i.e., 20 samples per class for training, 10 for testing). This random splitting process was repeated 10 times to generate independent training-testing splits. The model was trained and evaluated on each split, and the final classification accuracy is reported as the average performance over these 10 trials, accompanied by the standard deviation. This procedure reduces the influence of random data allocation on evaluation reliability. Partial samples of the SVM training and test data are provided in Table 4.

**Table 4:** The partial SVM training and test data.

Category	Range	Ringing count	Duration	Energy	Rise count	Rise time	RMS	ASL
1	85.6	1606	74,837	22464.3982	84	2006.5	0.761	49.5
1	83.8	1258	50,542	10079.3015	116	2116	0.632	46
1	83.8	1525	69,005	16824.2737	64	1958.5	0.624	47.7
1	83.9	1073	48143.5	11045.0485	92	2020	0.733	47.2
1	82.9	1381	55,881	9138.7009	56	1945.5	0.503	44.3
2	80.1	8086	138328.5	100970.4834	3510	37,337	1.206	57.3
2	82	11,282	195,532	138810.8475	7832	87377.5	1.321	57
2	77	8819	188,334	96524.1531	5171	92933.5	0.888	54.2
2	72	8323	148967.5	49133.5358	4917	65165.5	0.531	50.4
2	77.1	12,334	196,223	93520.4971	8038	96,712	0.823	53.6
3	89.8	4455	103773.5	118017.1829	59	474.5	2.503	61.1
3	85.8	4157	91,367	68001.5442	61	473.5	1.578	57.4
3	85.3	4464	90,805	70743.3914	66	473.5	1.654	57.8
3	86.5	4986	94894.5	89484.1476	812	7328	1.976	59.5
3	86.4	4456	85,698	67500.8041	67	473.5	1.663	57.9
1	87.9	1791	84824.5	28623.2269	61	1972	0.93	50.6
1	87.8	1696	80,705	27050.116	62	1983	0.912	50.5
2	81.3	10,798	176,710	106503.9536	6282	74,547	1.068	55.6
2	81.7	10,451	159592.5	111884.9625	5241	51,598	1.205	56.9
3	83.8	4783	85828.5	54494.8898	68	474	1.33	56.1
3	89.5	4963	117936.5	118972.7081	482	17246.5	2.391	60.1

**Fig. 9:**
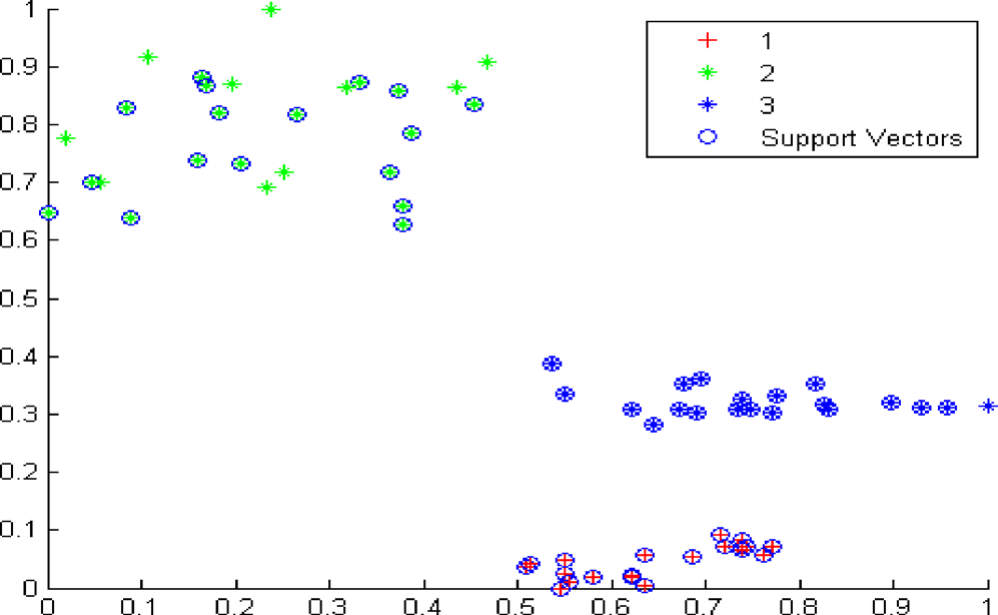
Cartogram of classification results under noise-free conditions.

Figure 9 presents the classification results of the SVM model under noise-free conditions. Using ten sets of normalized eigenvalues per leakage type as input, all sixty test samples were correctly classified, yielding an accuracy of 100% under ideal laboratory conditions, as summarized in Table 5.

**Fig. 10:**
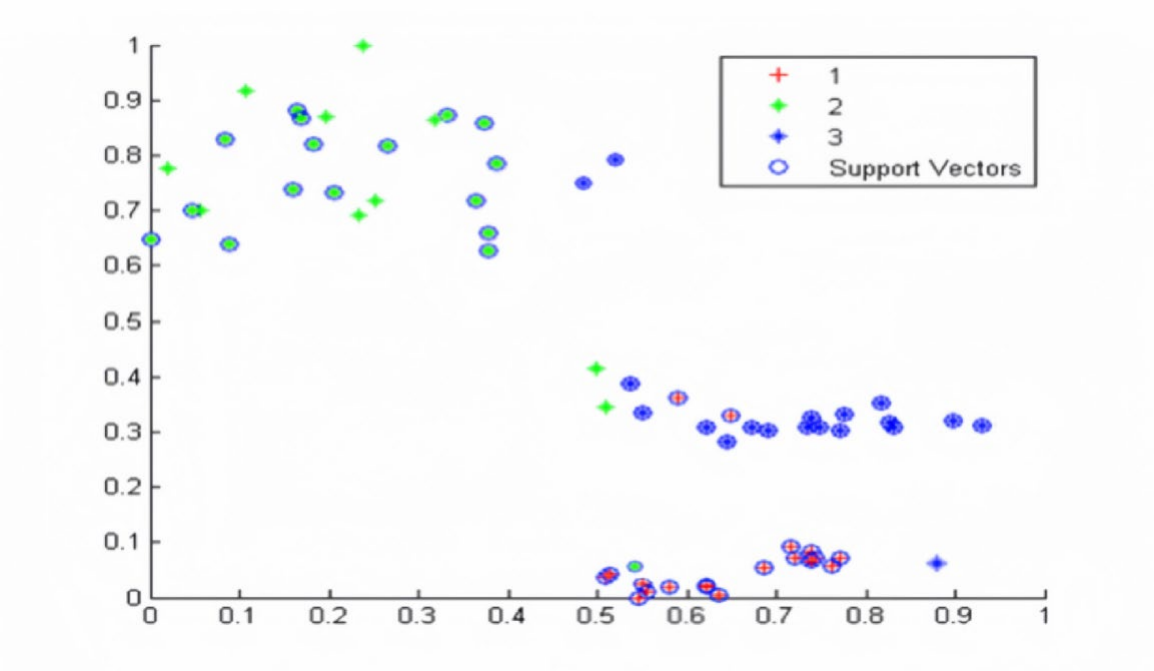
Cartogram of classification results under high-noise conditions.

Figure 10 displays the classification performance under high-noise simulated environments. When evaluated with the same feature input strategy, the model maintained robust performance, correctly classifying most test samples. Specifically, accuracies of 92.3% for spot leakage, 88.1% for fracture leakage, and 85.4% for explosion-induced leakage were achieved, as detailed in Table 5.

The proposed enhanced SVM framework was evaluated against a well-tuned traditional SVM model, which serves as the baseline. This baseline model employed a single RBF kernel, and its hyperparameters (penalty factor C and kernel parameter γ) were optimized using the same grid search and cross-validation procedure described in Sect. 3.4. This ensures a fair comparison, as the baseline represents a strong, conventionally optimized SVM. The experimental results indicate that our method, integrating multi-domain feature fusion, dynamic kernel selection, and boundary sample weighting, achieves a performance improvement of 12–15% over this strong baseline under high-noise conditions, highlighting the efficacy of the proposed enhancements.

**Table 5:** The recognition rate of the leakage of AE signal.

Leakage AE signal type	Recognition accuracy under noise-free conditions (mean ± SD %)	Recognition accuracy under high-noise conditions (mean ± SD %)
Spot leakage	100.0 ± 0.0	92.3 ± 1.5
Fracture leakage	100.0 ± 0.0	88.1 ± 2.0
Explosion tube leakage	100.0 ± 0.4	85.4 ± 2.2

To further evaluate the performance of the proposed method, we compared it with several widely-used machine learning classifiers, including Decision Tree (DT), Random Forest (RF), AdaBoost, and k-Nearest Neighbors (k-NN). All competing models were trained and tested on the same dataset partitions, with their hyperparameters optimized through a grid search procedure. As summarized in Table 6, the proposed enhanced SVM framework achieves the highest classification accuracy under high-noise conditions, outperforming all conventional machine learning models included in the comparison.

**Table 6:** Comparison of recognition accuracy under high-noise conditions with other machine learning methods.

Method	Spot Leakage(%)	Fracture Leakage(%)	Explosion Tube Leakage(%)	Average(%)
Proposed enhanced SVM	92	88	85	88.3
Traditional SVM (RBF)	80	76	72	76.0
Decision tree	85	79	74	79.3
Random forest	88	82	78	82.7
AdaBoost	87	81	77	81.7
k-NN	83	75	70	76.0

While deep learning methods have achieved remarkable performance in data-rich scenarios, they often encounter challenges such as overfitting and high computational demands in small-sample settings like ours, which comprises only 90 samples in total. To empirically validate this limitation, we implemented a simple 1D convolutional neural network (1D-CNN) as a comparative baseline.

The 1D-CNN model exhibited limited generalization capability, achieving an average accuracy of only 78.5% under high-noise conditions, which is significantly lower than the 88.3% attained by our proposed method. This outcome underscores the practical advantage of our enhanced SVM framework, which is explicitly designed for robustness and efficiency with limited data, making it particularly suitable for industrial applications where collecting large volumes of labeled leakage data is often infeasible.

#### Statistical validation

To rigorously evaluate model performance given the limited dataset size, we employed a 5-fold cross-validation strategy in addition to the hold-out test. The dataset was randomly divided into 5 folds, each containing representative samples from all three leakage types. In each iteration, the model was trained on four folds and validated on the remaining one, with this process repeated five times. The final classification accuracies presented in Table 4 represent the mean values across all folds, with standard deviations indicated in parentheses.

Furthermore, a paired t-test was performed to compare the classification performance between the proposed enhanced SVM and the traditional SVM under 70 dB noise conditions. The results demonstrated that the performance improvement achieved by our method is statistically significant (*p* < 0.01).

### Model training and parameter optimization

The SVM models, including both linear and RBF kernels, were implemented using the LIBSVM toolbox in MATLAB. To ensure optimal performance, hyperparameters specifically the penalty factor C and the RBF kernel parameter γ, were optimized via grid search combined with 5-fold cross-validation. The search ranges were set as C ∈ [2^− 5^,2^− 3^,…,2^15^] and [2^− 5^,2^− 3^,…,2^15^] and γ ∈ [2^− 15^,2^− 13^,…,2^3^]. The parameter combination achieving the highest average classification accuracy on the validation folds was selected for the final model.

### Limitations of noise simulation and industrial applicability

The high-noise condition (70 dB) in this study was simulated by adding Gaussian white noise within the 20–100 kHz band, which effectively represents a common type of broadband background interference in industrial settings. However, it should be noted that real-world boiler environments may contain additional noise sources, such as periodic vibrations from rotating machinery, electromagnetic interference from power equipment, and impulse noise from valve operations. These components could exhibit non-stationary and non-Gaussian characteristics, which were not explicitly modeled in the present experiments.

The choice of Gaussian white noise was motivated by its ability to provide a controllable and reproducible benchmark for evaluating model robustness. Future work will extend the noise model to include multi-component and non-stationary interference, thereby further validating the proposed framework under more realistic industrial acoustic conditions.

## Conclusions and future research

This study presents an enhanced SVM framework integrated with AE technology to address the challenges of pipeline leakage detection in boiler energy systems under constrained sample sizes and noisy industrial environments. Experimental results validate the effectiveness of the proposed approach, achieving 100% classification accuracy for spot leakage, fracture leakage, and explosion tube leakage under ideal laboratory conditions. Under simulated high-noise environments, the model maintains robust performance, with accuracies of 92.3%, 88.1%, and 85.4% for the three leakage types, respectively, representing a 12–15% improvement over traditional SVM. The success of the framework stems from three key innovations:

1. Multi-Domain Feature Fusion for Comprehensive Characterization. By integrating eight time-domain and frequency-domain parameters including Range, Energy, and RMS, we constructed a discriminative feature vector that captures both transient and spectral characteristics of leakage signals. This multi-domain representation enhances separability among leakage types and improves robustness against noise compared to single-domain features;
2. Adaptive Model Complexity via Spectral Sparsity-Guided Kernel Selection.Conventional SVM models employ a fixed kernel, which may not optimally represent the diverse spectral properties of different leak types. Our dynamic kernel selection mechanism, driven by a spectral sparsity index (SSI), adaptively chooses between linear and RBF kernels. This ensures an appropriate trade-off between model complexity and generalization capability for each signal category.
3. Enhanced Noise Robustness through Margin-Based Boundary Sample Weighting. To mitigate the effect of noise-corrupted samples near the decision boundary, a sample weighting strategy is introduced that assigns higher weights to low-margin instances during training. This directs the SVM to prioritize boundary clarity and improves resilience to noisy samples, accounting for the observed 12–15% performance gain in high-noise settings.

In summary, the proposed framework combines enhanced feature representation, adaptive model structure, and explicit noise handling to form an integrated solution that narrows the gap between theoretical model capacity and practical deployment needs in industrial leakage diagnostics.

Future research should prioritize the following directions to advance pipeline leak detection technology:

1. Integration with Deep Learning. Hybrid architectures combining the interpretability and sample efficiency of our enhanced SVM with the hierarchical feature extraction capability of convolutional neural networks could be explored for more complex multi-class scenarios;
2. Adaptive Noise Modeling and Enhanced Robustness. Future studies should focus on developing adaptive noise models that go beyond Gaussian white noise to incorporate realistic, non-stationary interference (e.g., periodic mechanical vibrations, electromagnetic pulses). Techniques such as online noise characterization and adversarial training could be integrated to further improve model robustness in highly dynamic industrial environments;
3. Online Learning and Threshold Adaptation. Implementing an online learning mechanism for the spectral sparsity index (SSI) threshold would allow the dynamic kernel selection to self-adapt to changing signal characteristics and noise profiles over time, enhancing long-term deployment viability without frequent manual recalibration;
4. Real-Time Deployment and Edge Computing. Optimization of the framework for deployment on edge computing platforms is essential to achieve low-latency, online leakage monitoring, enabling rapid response in field applications;
5. Expanded Validation and Generalizability. Comprehensive testing on larger-scale, diverse pipeline systems (e.g., coal-fired, biomass boilers) under varied operational conditions is crucial to validate the method’s generalizability and industrial readiness.

In conclusion, this study establishes a foundation for intelligent leakage detection in energy systems, effectively bridging theoretical innovation with industrial practicality. By addressing the outlined research challenges‒particularly in adaptive noise handling and online model adjustment‒the proposed framework demonstrates significant potential to enhance operational safety, reduce economic losses, and promote the adoption of AI-driven predictive maintenance strategies throughout the energy sector.

## Supplementary Information

Below is the link to the electronic supplementary material.

Supplementary Material 1

## Data Availability

The data presented in this study are available on request from the corresponding author due to commercial application data.
