# Supplementary material for: A pipeline leakage detection method for boiler energy operation system using enhanced SVM-based acoustic emission technology
Source: Sci Rep. 2026 Mar 7;16:12520. doi: 10.1038/s41598-026-42769-5 (PMC13087036; doi:10.1038/s41598-026-42769-5)
Supplement: Supplementary file 1 — Supplementary Material 1 [file 41598_2026_42769_MOESM1_ESM.doc]

Supporting Information

A pipeline leakage detection method for boiler energy operation system using enhanced SVM-based acoustic emission technology

Tianlong Yuan1,2*   Xiaofei Zhang3   Qian Zhang3   Minghang Tan1,2     

1. Liaoning Provincial Engineering Research Center for High-Value Utilization of Magnesite , Yingkou 115014, China
2. Liaoning Provincial Key Laboratory of Energy Storage and Utilization, Yingkou Institute of Technology,Yingkou, China
3.State Grid Yingkou Electric Power Supply Company, Yingkou, China
* Correspondence: yuantianlong@yku.edu.cn.  


List of Contents

FIGURE S1  Cartogram of classification results under noise-free conditions
FIGURE S2  Cartogram of classification results under high-noise conditions
TABLE S1  The partial SVM training and test data


FIGURE S1  Cartogram of classification results under noise-free conditions


FIGURE S2  Cartogram of classification results under high-noise conditions


data = readtable("source data.xlsx");
x = table2array(data(:, 2)); 
y = table2array(data(:, 3)); 
g = table2array(data(:, 1)); 
unique_groups = unique(g);
num_groups = length(unique_groups);
colors = 'rgb'; 
markers = '+**'; 
gscatter(x, y, g, colors, markers);
sv_indices = table2array(data(:,4)) == 1;
sv_plot = plot(x(sv_indices), y(sv_indices), 'bo', 'MarkerFaceColor', 'none', 'DisplayName', 'Support Vectors');
legend_text = [cellstr(num2str(unique_groups)); 'Support Vectors'];
legend(legend_text);


TABLE S1  The partial SVM training and test data

Category	Range	Ringing count	Duration	Energy	Rise count	Rise time	RMS	ASL	
1	85.6	1606	74837	22464.3982	84	2006.5	0.761	49.5	
1	83.8	1258	50542	10079.3015	116	2116	0.632	46	
1	83.8	1525	69005	16824.2737	64	1958.5	0.624	47.7	
1	83.9	1073	48143.5	11045.0485	92	2020	0.733	47.2	
1	82.9	1381	55881	9138.7009	56	1945.5	0.503	44.3	
1	83.2	1791	84824.5	28623.2269	61	1972	0.93	50.6	
1	83.4	1696	80705	27050.116	62	1983	0.912	50.5	
1	83.4	1285	76894	17852.2187	68	2024	0.845	49.3	
1	83.5	1356	68951	17854.2145	64	2108	0.689	48	
1	83.9	1359	77412	24576.2589	59	1997	0.652	47.8	
1	83.2	1541	73524	22634.2354	52	1966	0.631	46.5	
1	84.1	1602	66521	19875.2569	88	1958	0.754	47.2	
1	84.6	1713	68412	16589.2578	82	2004	0.763	47.4	
1	84.3	1695	58652.5	22583.7456	77	2012	0.724	47.6	
1	84.4	1425	54921	14526.3698	73	1988	0.689	46.8	
1	83.9	1434	66524	18975.2367	99	1974	0.845	49.6	
1	84.6	1589	67149	17854.5819	101	1955.5	0.94	50.2	
1	85.1	1654	77541.5	26587.5278	91	2089	0.921	50.1	
1	84.7	1325	71548.5	22574.2597	64	2045	0.822	49.1	
1	84.3	1187	54078	14587.5839	63	1972.5	0.864	48.6	
1	87.9	1791	84824.5	28623.2269	61	1972	0.93	50.6	
1	87.8	1696	80705	27050.116	62	1983	0.912	50.5	
1	87.2	1784	78564	26528.556	73	1999	0.902	50.3	
1	87.9	1852	77421	28596.594	69	2102	0.858	49.3	
1	88.6	1741	76258	25873.257	88	2045	0.669	47.9	
1	88.1	1952	68952	18995.541	81	1987	0.745	47.1	
1	88.4	1842	78468	19541.259	77	1975	0.895	49.9	
1	88.9	1773	75248	23659.489	69	1947	0.952	50.9	
1	88.8	1698	76982	26932.256	57	1992	0.758	47.8	
1	89.2	1856	73258	22358.369	76	1996	0.713	47.5	
2	80.1	8086	138328.5	100970.4834	3510	37337	1.206	57.3	
2	82	11282	195532	138810.8475	7832	87377.5	1.321	57	
2	77	8819	188334	96524.1531	5171	92933.5	0.888	54.2	
2	72	8323	148967.5	49133.5358	4917	65165.5	0.531	50.4	
2	77.1	12334	196223	93520.4971	8038	96712	0.823	53.6	
2	74.2	9845	152498	84569.4512	4589	68524	0.633	48.9	
2	73.9	8547	168579	85498.2354	8524	75456	0.854	49.1	
2	75.3	8654	178956	79546.2354	7854	85245	0.984	47.9	
2	77.6	8215	154789	88412.2596	6982	69852	1.014	52.9	
2	79.8	7984	145896	90214.2564	5874	77854	1.128	49.8	
2	80.2	8857	158713	87546.2598	4996	78235.5	0.954	48.5	
2	72.6	8945	185565	82564.2587	5862	85247	0.874	54.3	
2	70.5	9425	198552	78925.3657	5367	89654	0.784	54	
2	71.9	9746	182289	102587.2579	5897	77451	0.845	50.2	
2	74.9	9354	175846.5	110246.5812	6698	68523.5	0.981	52.4	
2	73.8	9854	168957	96524.2531	6258	60524	1.156	56.6	
2	77.6	10145	172258	92548.5812	7759	58741	1.089	49.5	
2	78.4	9356	185247	99854.2547	8205	62587	1.256	52.3	
2	73.7	9287	175489	91257.2793	8024	79548	0.993	54.5	
2	74.7	9667	169854	88524.5861	6412	84254.5	0.901	51.2	
2	81.3	10798	176710	106503.9536	6282	74547	1.068	53.6	
2	81.7	10451	159592.5	111884.9625	5241	51598	1.205	54.9	
2	82.5	8657	172289	895243.5247	6258	69524	1.025	55.2	
2	84.2	8745	185846.5	845247.5689	6258	89564.5	0.952	49.5	
2	78.9	9625	158957	92548.2186	7129	67821	0.965	51.3	
2	79.6	9246	162258	96542.1897	8895	45986	0.924	56.2	
2	81.5	9154	195247	93214.1297	8134	69862	1.026	54.2	
2	83.6	9654	165489.5	112586.2356	6462	85473.5	0.922	53.6	
2	81.9	9145	179854	99856.2579	9282	75831	0.824	55.1	
2	79.2	10356	168710	90254.5627	7359	88563	1.054	58.4	
3	89.8	4455	103773.5	118017.1829	59	474.5	2.503	61.1	
3	85.8	4157	91367	68001.5442	61	473.5	1.578	57.4	
3	85.3	4464	90805	70743.3914	66	473.5	1.654	57.8	
3	86.5	4986	94894.5	89484.1476	812	7328	1.976	59.5	
3	86.4	4456	85698	67500.8041	67	473.5	1.663	57.9	
3	84.2	4652	84259	84523.2354	56	493.6	1.789	58.6	
3	81.6	4875	75962	82154.2356	89	785.4	1.892	57.2	
3	84.9	4125	68952.5	78953.2145	78	725.6	1.523	54.9	
3	87.2	4658	87421	72459.2397	95	652.8	1.458	52.6	
3	86.5	4258	66985	56894.2578	85	469.6	1.698	53.2	
3	82.6	4523	72458	66985.2563	458	6852	1.827	60.8	
3	84.9	4236	96523	79852.2547	58	525.9	1.456	54.1	
3	88.1	4229	84789.5	89562.4789	96	745.2	1.826	53.8	
3	87.6	4865	94258	102458.2586	52	482.6	1.956	56.1	
3	83.6	4986	89571	101452.5652	54	493.6	1.712	54.6	
3	84.7	4752	86582	99852.3652	76	589.3	1.689	57.2	
3	82.5	4258	92524	92548.2568	72	623.7	1.556	56.5	
3	83.7	4785	81257	89547.2589	463	6952	1.986	61.9	
3	84.5	4985	88524	95368.2574	55	485.2	1.336	58.1	
3	82.9	4655	10258.5	66587.2593	87	493.6	1.82	57.6	
3	83.8	4783	85828.5	54494.8898	68	474	1.33	56.1	
3	89.5	4963	117936.5	118972.7081	482	17246.5	2.391	60.1	
3	88.7	4857	95286	85225.2587	54	489.6	1.698	58.3	
3	86.9	4556	85754	77489.2569	98	824.5	1.854	57.4	
3	89.2	4368	89652	68563.2587	95	865.2	1.652	56.6	
3	87.5	4298	97523.5	76352.2541	66	589.6	1.875	56.3	
3	84.7	4189	93254.5	88523.6985	86	691.2	1.785	58.9	
3	86.9	4853	11258.5	96358.2549	73	925.5	1.654	59.2	
3	85.5	4578	99862	95852.3658	485	16528.6	2.456	63.2	
3	81.8	4921	86526	68952.3658	57	486.5	1.524	57.3	
